# A novel mutation in the KLHL17 gene is associated with neurodevelopmental disorders

**DOI:** 10.1016/j.gendis.2025.101528

**Published:** 2025-01-10

**Authors:** Meng Ao, Shunxiang Zhang, Yun Ouyang, Shucong Li, Heqian Ma, Meizhen Guo, Xuelin Dai, Qianhui Xia, Xiaoying Zhang

**Affiliations:** a The School of Public Health, Guilin Medical University, Guilin, Guangxi 541100, China; b The Guangxi Key Laboratory of Environmental Exposomics and Entire Lifecycle Heath, Guilin, Guangxi 541199, China; c Guangxi Health Commission Key Laboratory of Entire Lifecycle Health and Care, Guilin, Guangxi 541199, China

Kelch-like family member 17 (KLHL17) is predominantly expressed in the brain and plays a crucial role in neuronal development and function, deletions and/or mutations in KLHL17 have been linked to neurodevelopmental disorders in humans, *e.g.*, intellectual disability, autism spectrum disorder, and infantile spasms, but the etiology and pathogenesis remain largely enigmatic.^1^^,^^2^ As a member of the family of the Kelch proteins, KLHL17 contains an N-terminal BTB/POZ domain followed by a BACK domain and four to six tandem Kelch motifs at the C-terminal region (Fig. S1A).^1^^,^^3^ Previously, we identified a novel *de novo* variant in KLHL17 (c.701C > T; p. P234L) in a cohort of 225 Chinese children with developmental delay/intellectual disability based on whole-exome sequencing (1/225), the mutation located in the BACK domain, a very high conversed region (Fig. S1B), and the affected boy presented with developmental delay, intellectual disability, hypotonia, and abnormal brainstem auditory evoked potential signal.^4^ The finding may offer a new clue to investigate the molecular pathogenesis of KLHL17 gene in neurodevelopmental disorders.

Firstly, online REVEL (https://sites.google.com/site/revelgenomics/) and CADD (https://cadd.gs.washington.edu/) tools were employed to predict pathogenicity for KLHL17^P234L^ variant, and high REVEL and CADD scores (0.907 and 25.9, respectively) indicated that the mutation was associated with a high probability of pathogenicity. Subsequently, the P234L amino acid change was precisely predicted by AlphaFold 3 and PyMOL 2.3.0. As shown in Figure 1A, the mutated L234 establishes a new hydrogen band with G237, and local charge distribution differs greatly between KLHL17^wt^ and KLHL17^P234L^, suggesting that such mutation is likely to affect the conformational characterization and protein–protein interaction, including enzyme/substrate specificity, affinity, and activity.

**Figure 1:**
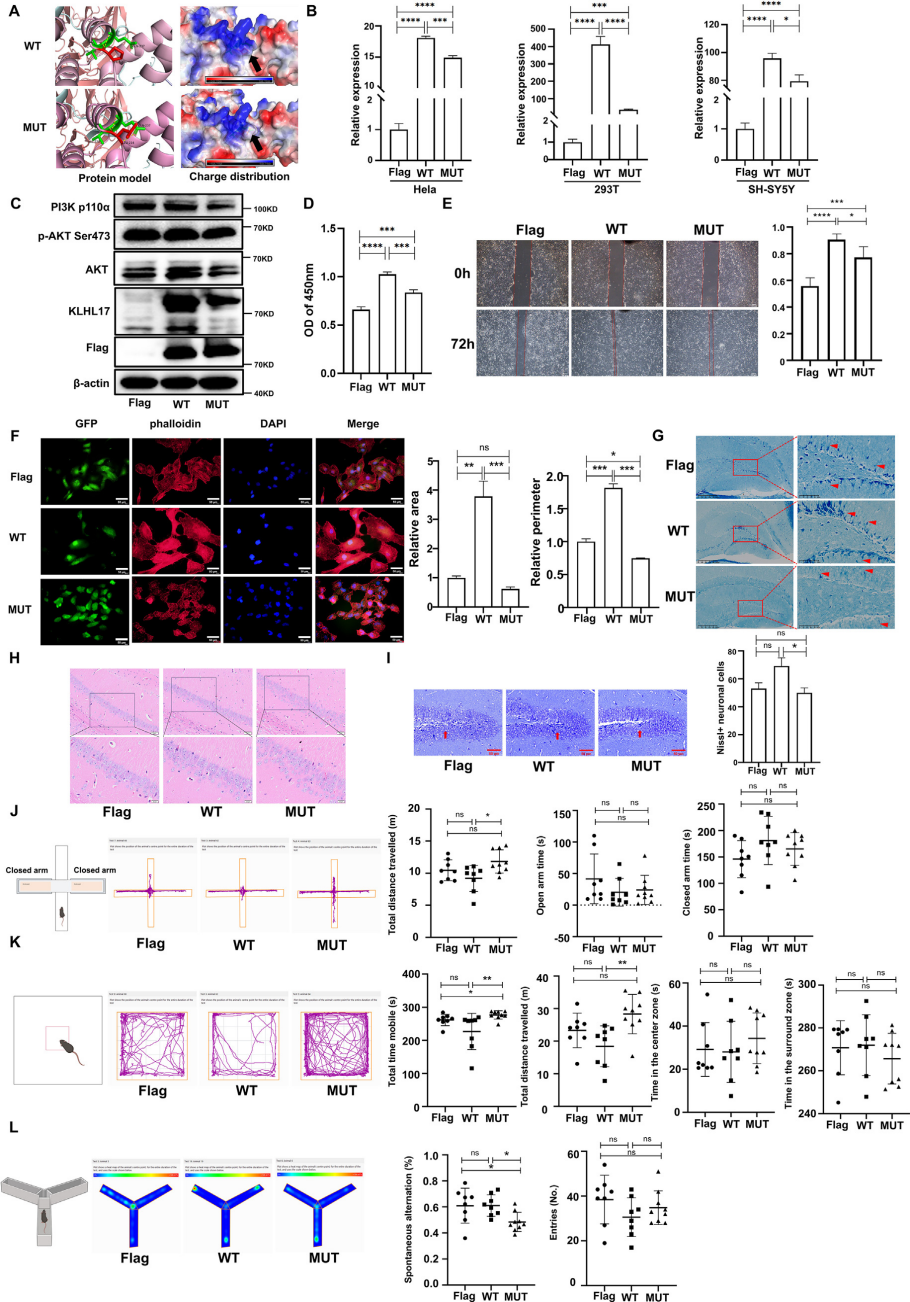
A novel mutation in the KLHL17 gene is associated with neurodevelopmental disorders. **(A)** KLHL17's structure and homology modeling of KLHL17^P234L^ were predicted using AlphaFold 3 (https://golgi.sandbox.google.com/) and were visualized in 3D using PyMOL software 2.3.0. **(B)** Quantitative real-time PCR analysis of KLHL17 expression in Hela, 293T, and SH-SY5Y cells transfected with different vectors for 72 h. **(C)** Western blot analysis of the expression of KLHL17, PI3K, AKT, and p-AKT in 293T cells transfected with different vectors for 72 h. **(D)** CCK8 assay showed the proliferation ability of SH-SY5Y cells (72 h after transfection). **(E)** The wound healing assay showed the migration ability of SH-SY5Y cells. Bar: 200 μm. Vector doses in (B–D) were the same. **(F)** KLHL17 altered cell morphology. Left: representative images of stable Hela cells (bar: 50 μm). Right: quantification of cell area and perimeter. **(G–L)** Animal model: axon myelination of mouse hippocampal neurons was investigated by Luxol fast blue staining (DG area; the bar is 50 μm for the right and 200 μm for the left) (G); KLHL17^P234L^-administrated mice showed demyelination, while KLHL17^wt^ and vector control mice showed normal myelination (red arrowhead); hippocampal neuronal morphology and Nissl bodies in the cytoplasm were visualized by hematoxylin and eosin staining (CA1 area, bar: upper: 50 μm, below: 20 μm) (H) and Nissl staining (DG area; bar: 50 μm) (I); hematoxylin and eosin staining and Nissl staining showed that the neurons in the hippocampal CA1 or DG area of the mice in the KLHL17^wt^ and vector control groups were neatly arranged, with clear nucleoli, while the neurons in KLHL17^P234L^ group were sparsely arranged, with nuclear pyknosis and enlarged intercellular space; and Nissl staining revealed that KLHL17^P234L^-administrated group had the least number of Nissl-positive neurons (I, right). Motor, learning, memory, and cognition were evaluated by a series of behavior assays, including the elevated plus maze test (EPMT) (J), open field test (OFT) (K), and Y-Maze test (YMZT) (L). KLHL17^P234L^-administrated mice exhibited hyperactivity (J, K) and impaired spatial learning and memory (L), compared with KLHL17^wt^-administrated mice; additionally, KLHL17^P234L^ mice did not exhibit anxiety-like behavior, *i.e.*, the three groups of mice spent comparable time in surround or center zone in OFT (K), and spent equivalent time in the open or closed arms in EPMT (J). Flag, 3xflag-tagged empty vector control; WT, 3xflag-tagged KLHL17^wt^ vector; MUT, 3xflag-tagged KLHL17 ^P234L^ vector. Data are presented as mean ± standard deviation. To compare multiple groups, the data were analyzed by one-way analysis of variance (ANOVA) followed by Tukey's post hoc test if data were normally distributed or by the Kruskal–Wallis test followed by Mann–Whitney U test if the data were non-normally distributed. ∗*P* < 0.05, ∗∗*P* < 0.01, ∗∗∗*P* < 0.001, ∗∗∗∗*P* < 0.0001; ns, no significance.

Because missense mutation may cause haploinsufficiency by affecting mRNA and/or protein expression, we sought to evaluate the impact of the identified KLHL17^P234L^ mutation on KLHL17 mRNA and/or protein expression. To this end, a fused N-terminal 3xflag-tagged KLHL17^wt^, KLHL17^P234L^, or empty vector was expressed in human Hela, 293T, or SH-SY5Y cells with similar transfection efficiency. We found that the KLHL17^P234L^ overexpression cells displayed significantly lower KLHL17 mRNA (Fig. 1B) and protein (Fig. 1C) levels when compared with KLHL17^wt^ overexpression cells (Fig. 1B, C). Then, we further examined cell proliferation and migration by CCK8 and wound healing test, we found that overexpression of ectopic KLHL17^wt^ in SH-SY5Y cells could promote cell proliferation and migration (Fig. 1D, E), the phenomena were also observed in other cell lines, such as A549, H299, H460, and SK cells,^3^ however, the promotion was significantly inhibited by KLHL17^P234L^ mutation (Fig. 1D, E). Meanwhile, cell morphology and F-actin organization were examined among Hela cells with stable expression of 3xflag-tagged KLHL17^wt^, KLHL17^P234L^, or empty vector control. Intriguingly, we found that stable cells expressing KLHL17^wt^ extended well, whereas KLHL17^P234L^-expressiing cells were much smaller (Fig. 1F, left); quantification of cell area and cell perimeter based on F-actin signal further substantiated that KLHL17^P234L^-expressiing cells were significantly smaller than KLHL17^wt^-expressiing cells (Fig. 1F, right). Therefore, these data indicate that this mutation can result in decreased gene expression and abnormal morphology and functions of cells.

To eliminate the possibility that the variant's effect on cell function is due to haploinsufficiency, we doubled the dosage of the KLHL17 ^P234L^ vector during the transient transfection, ensuring that mRNA and protein expression levels of KLHL17 in the KLHL17^P234L^-exprssing cells matched or exceeded that in the KLHL17^wt^-exprssing cells (Fig. S2A, B). Furthermore, we found that compared with vector control cells, both KLHL17^wt^ and KLHL17^P234L^ overexpression cells significantly increased cell proliferation and migration, and KLHL17^P234L^ cells still displayed less cell proliferation and migration levels than KLHL17^wt^ cells (Fig. S2C, D). Together, the findings imply that apart from haploinsufficiency, KLHL17^P234L^ mutation can influence cell biological function.

The phosphoinositide 3-kinase (PI3K)/protein kinase B (AKT) pathway emerges as a pivotal signal pathway implicated in cell proliferation, migration, differentiation, and metabolism; it has been considered a key driver of neurogenesis and its dysregulation has been implicated in the pathogenesis of various neurodevelopmental disorders, including autism spectrum disorder and intellectual disability.^5^ In our study, we found that overexpression of KLHL17 induced by KLHL17^wt^ plasmid elevated expression of PI3K-p110 and phosphorylated AKT, whereas KLHL17^P234L^ suppressed the activation (Fig. 1C). Furthermore, no significant differences were found among the three groups regarding cell proliferation after PI3K inhibitor GDC-0941 treatment (Fig. S3), indicating the increased proliferation levels induced by KLHL17^wt^ and KLHL17^P234L^ could be inhibited by attenuating the activation of PI3K/AKT pathway. Together, these findings suggest that the effect of KLHL17 and KLHL17^P234L^ in regulating cellular biological functions is accomplished through, or at least partly through, the activation of the PI3K/AKT pathway, thereby making it an attractive target for molecular pathogenesis and therapeutic intervention studies.

Next, to investigate the role of KLHL17^P234L^ in brain function *in vivo*, newborn mice were administered with different lentivirus (KLHL17^wt^, KLHL17^P234L^, or empty vector control) with the same injection dose (2 μL lentivirus; titer: 2 × 10^5^ TU/mL) via intracerebroventricular injection; 120 days later, histological analysis and a series of behavior assays were performed (more detail is shown in Supplementary Material; Fig. S4). As expected, we detected flag-positive signals in the mouse hippocampus of all three groups (Fig. S5), indicating the lentivirus had successfully delivered the plasmid DNA in the hippocampus; and the KLHL17 mRNA expression in the KLHL17^wt^-administrated mice was significantly higher than that in KLHL17^P234L^-administrated mice and vector control mice (Fig. S6). We examined axon myelination of hippocampal neurons by Luxol fast blue staining. Intriguingly, we found that cells in the hippocampal DG area of KLHL17^P234L^-administrated mice were myelination-deficient, while vector control and KLHL17^wt^-administrated mice showed normal myelination (Fig. 1G). KLHL17^P234L^-induced neuronal loss was obvious in the hippocampal CA1 or DG area in the KLHL17^P234L^-administrated mice (Fig. 1H, I), whereas vector control and KLHL17^wt^-administrated mice showed normal neuronal cells with clear nucleoli and neatly arranged neurons in hippocampal areas (Fig. 1H, I). Moreover, mouse behavioral experiments (Fig. 1J–L) revealed that compared with KLHL17^wt^ mice, KLHL17^P234L^ mice exhibited significantly longer moving distances in the elevated plus maze test and the open field test (Fig. 1J, K), less spontaneous alternation percentage in Y-maze test (Fig. 1L), which suggested that KLHL17^P234L^ could induce hyperactivity, spatial learning, and memory impairment *in vivo*. Additionally, we did not find that KLHL17^P234L^-administrated mice exhibited anxiety-like behavior in this study (Fig. 1K–J). Consistently, Hu et al also found that compared with *Klhl17*^+/+^ mice, *Klhl17*^+/−^ mice displayed hyperactivity but no anxiety responses.^1^ Indeed, in several unrelated patients, deletion of different segments of chromosome 1p36 that include KLHL17 display a wide phenotypic spectrum, including developmental delay, mental retardation, and seizure/epilepsy.^2^ One conclusion could thus be that although the abnormal behavioral phenotypes in KLHL17^P234L^-administrated mice did not perfectly match human behaviors exhibited by the developmental delay/intellectual disability boy with KLHL17^P234L^ mutation, the subsequent behavioral and pathological findings in the KLHL17^P234L^-administrated mice were attributable to exogenous KLHL17^P234L^ expression, highlighting its potential value for academic research and clinical fields.

In conclusion, although the effect of KLHL17^P234L^ requires further in-depth study, for example, more molecular experiments are needed to identify the specific targets or interacting genes of KLHL17 in neurodevelopmental disorders, as well as more behavioral experiments across different time points to undertake more detailed analysis of the pathological effect of the P234L mutation on neuronal development *in vivo*, our findings furnish novel research cues for understanding the molecular pathogenesis and mechanism of KLHL17-related neurodevelopmental disorders.

## Ethics declaration

Human-related study was not performed in this study. For animal study, all experiments were performed with the approval of the Guilin Medical University Ethics Committee (No. GLMC202303105).

## Funding

This study was funded by the 10.13039/501100001809National Natural Science Foundation of China (No. 82160620), the 10.13039/501100004607Natural Science Foundation of Guangxi Province, China (No. 2023GXNSFAA026036), and the 10.13039/501100013254Guangxi College Students Innovation and Entrepreneurship Training Program (China) (No. S202310601164).

## CRediT authorship contribution statement

**Meng Ao:** Writing – original draft, Software, Methodology, Investigation. **Shunxiang Zhang:** Software, Investigation, Data curation. **Yun Ouyang:** Writing – original draft, Software, Data curation. **Shucong Li:** Visualization, Software. **Heqian Ma:** Visualization, Methodology. **Meizhen Guo:** Formal analysis. **Xuelin Dai:** Software. **Qianhui Xia:** Software. **Xiaoying Zhang:** Writing – review & editing, Project administration, Funding acquisition, Conceptualization.

## Conflict of interests

The authors declared no conflicting interests.

## Figures and Tables

**Figure S1:**
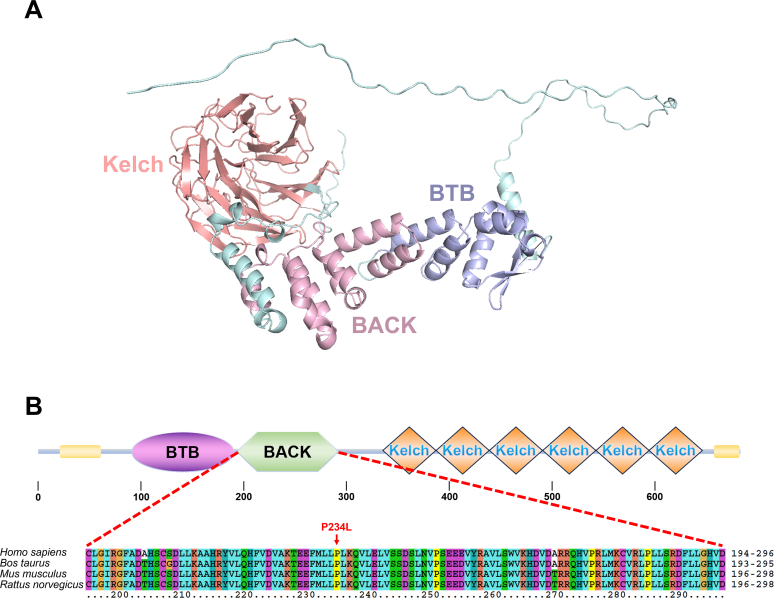
Computerized analysis of the KLHL17 structure. **(A)** 3D structure of KLHL17 using PyMOL software 2.3.0. **(B)** Schematic structure of KLHL17 and the mutation (red arrow) localizing in the BACK domain, a highly conserved region.

**Figure S2:**
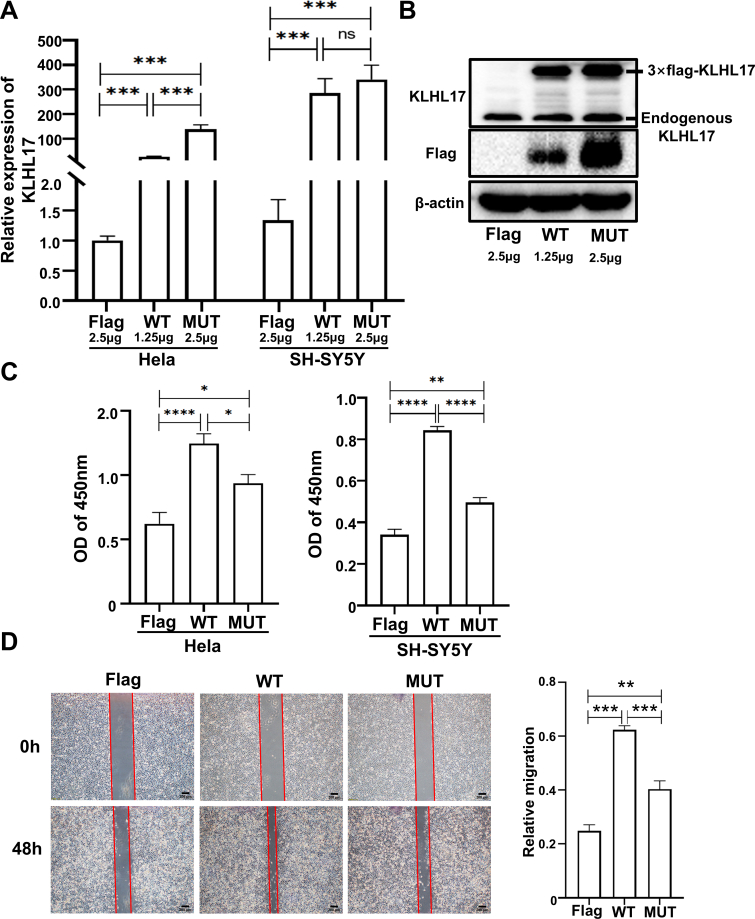
Levels of mRNA and protein expression of KLHL17 in KLHL17^P234L^ doubled transient transfection. **(A, B)** Quantitative real-time PCR (A) and western blot analysis (B) of KLHL17 expression in cells transfected with different vectors (72 h after transfection). **(C)** CCK8 assay showed the effects of KLHL17 overexpression with different vectors (72 h after transfection) on the proliferation ability of Hela and SH-SY5Y cells. **(D)** The wound healing assay showed the effects of KLHL17 overexpression with different vectors on the migration ability of Hela cells (bar: 200 μm). Flag, 3xflag-tagged empty vector control; WT, 3xflag-tagged KLHL17^wt^ vector; MUT, 3xflag-tagged KLHL17^P234L^ vector.

**Figure S3:**
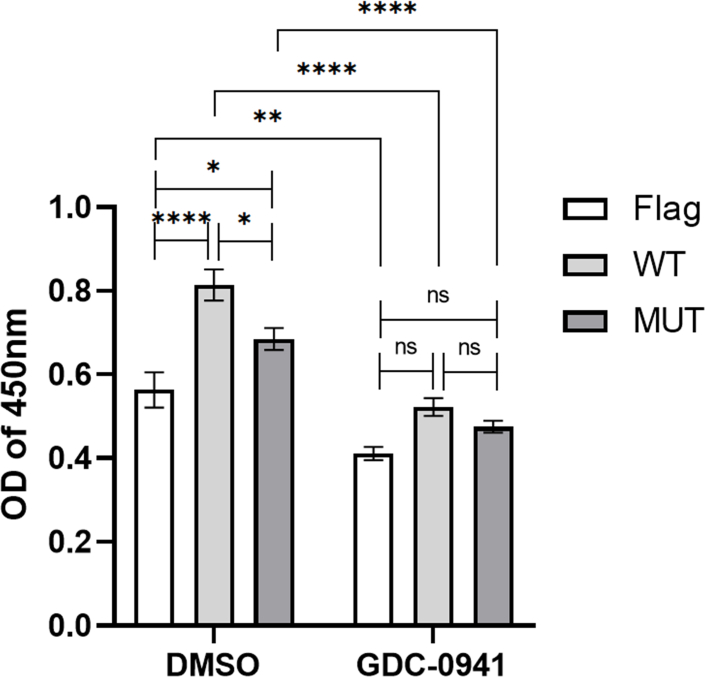
CCK8 assay showed the effects of small molecule inhibitor GDC-0941 on SH-SY5Y cell proliferation (72 h after transfection). Two-way ANOVA with Tukey's post hoc test for multiple comparisons was applied. ∗*P* < 0.05, ∗∗*P* < 0.01, ∗∗∗*P* < 0.001, ∗∗∗∗*P* < 0.0001; ns, no significance.

**Figure S4:**
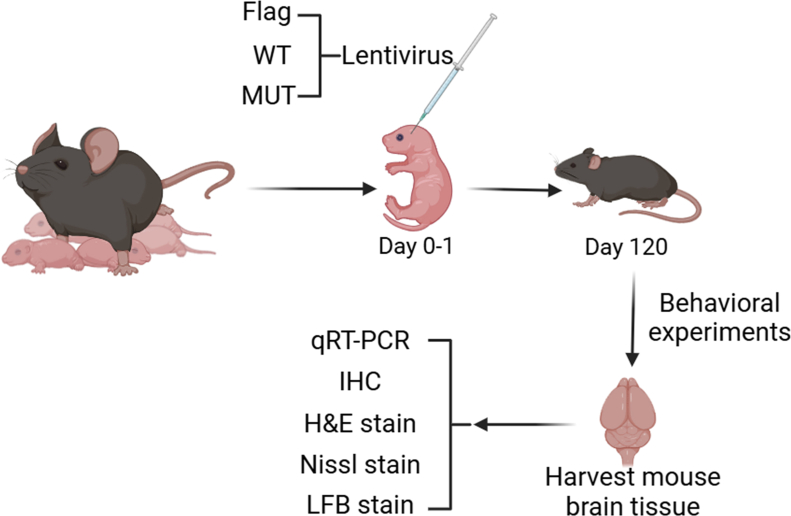
The schematic diagram of the animal experiment. Briefly, post-natal 0–1 day (P0–1) mice (1.2–1.5 g) were randomly divided into three groups (*n* = 9 per group): KLHL17^wt^ vector group, KLHL17^P234L^ vector group, and empty vector group; then, the mice were anesthetized on ice for 1–3 min, 2 μL lentivirus (titer: 2 × 10^5^ TU/mL) was slowly administered to newborn mice via intracerebroventricular injection. Notably, to simulate the actual situation of KLHL17^P234L^ expression *in vivo*, we did not adjust the lentivirus injection dose to make equal expression levels of KLHL17 and KLHL17^P234L^ in mice. All the newborn mice were examined daily to evaluate their survival status. 120 days later, exogenous human KLHL17 expression levels in the hippocampus region were detected by quantitative PCR and immunohistochemistry assay; hippocampal neuronal morphology was visualized by Luxol fast blue staining, hematoxylin & eosin staining, and Nissl staining; motor, learning, memory, and cognition were evaluated by a series of behavior assays. Flag, 3xflag-tagged empty vector-administrated mice; WT, 3xflag-tagged KLHL17^wt^ vector-administrated mice; MUT, 3xflag-tagged KLHL17^P234L^ vector-administrated mice. The figure was created with BioRender.com.

**Figure S5:**
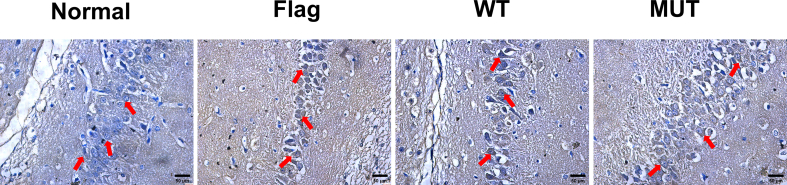
Immunohistochemical staining of Flag proteins in the hippocampal CA1 area of the mice intracerebroventricularly administrated with different lentivirus for 120 days. Normal, age-matched normal mice without any treatment; Flag, 3xflag-tagged empty vector-administrated mice; WT, 3xflag-tagged KLHL17^wt^ vector-administrated mice; MUT, 3xflag-tagged KLHL17^P234L^ vector-administrated mice.

**Figure S6:**
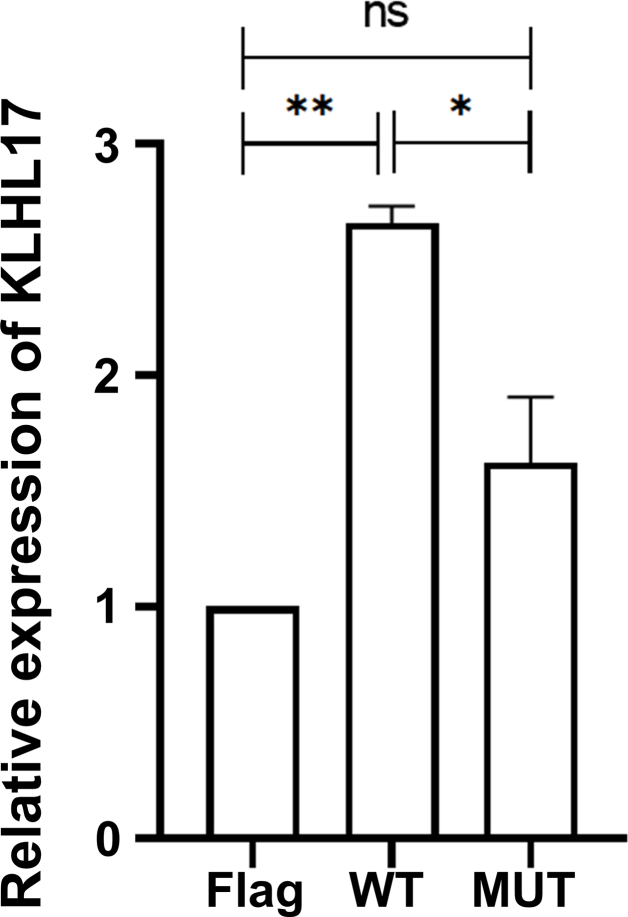
Quantitative real-time PCR for KLHL17 mRNA expression in the hippocampus of the mice intracerebroventricularly administrated with different lentivirus for 120 days. ∗*P* < 0.05, ∗∗*P* < 0.01; ns, no significance. Flag, 3xflag-tagged empty vector-administrated mice; WT, 3xflag-tagged KLHL17^wt^ vector-administrated mice; MUT, 3xflag-tagged KLHL17^P234L^ vector-administrated mice.

## Data Availability

The data used and/or analyzed in the study are included in the article or the Supplementary Material. Further inquiries can be directed to the corresponding author.
