# Supplementary material for: A novel mutation in the KLHL17 gene is associated with neurodevelopmental disorders
Source: Genes Dis. 2025 Jan 10;12(5):101528. doi: 10.1016/j.gendis.2025.101528 (PMC12164030; doi:10.1016/j.gendis.2025.101528)
Supplement: Multimedia component 1 [file mmc1.docx]

**Supplementary Material**

**Cells and cell culture**

HEK 293T, SH-SY5Y and Hela cells (purchased from Institute of Basic Medical Sciences Chinese Academy of Medical Sciences) were cultured in DMEM (Gibco-BRL, NY, USA), with 10% fetal bovine plasma (Gibco-BRL, NY, USA) at 37°C in 5% CO_2_.

**Plasmid construction and lentivirus production**

For 3xflag-tagged KLHL17 expression plasmid, full-length human KLHL17 (NM_198317) was PCR-amplifed and subcloned into lentiviral vector

pEB-3xflag-GFP(T2A)-PURO (Fitgene Biotech Co, China) digested by BamHI and EcoRI. To generate 3xflag-tagged mutated KLHL17 (c.701C>T) expression plasmid, mutated KLHL17 PCR product was synthesized based on the template of the KLHL17^wt^ pEB vector, then the mutated KLHL17^P234L^ vector was constructed by homologous recombination using PCR products and ClonExpress II one-step ligation kit (C112-2, VazyME, China). The correct base substitution was confirmed by DNA sequencing. All primers are listed in Table S1.

Lentivirus encoding KLHL17^wt^ (positive control), KLHL17^P234L^, or empty vector control (negative control) was produced by transfecting lentiviral-transfer vector (pEB-3xflag-KLHL17-WT-GFP(T2A)-PURO, pEB-3xflag-KLHL17-P234L -GFP(T2A)-PURO, or pEB-3xflag-GFP(T2A)-PURO) and packaging plasmids (pCMV-VSV-G and p8.9 NdeltaSB P90A) into 293T cells by lip 8000 (Beyotime, Shanghai, China). Supernant of cell cultures at 48 h and 72 h posttransfection was harvested and concentrated by PEG8000 (Solarbio, Beijing, China) precipitation; then the purified lentivirus titer was quantified by GFP expression assay ^1^.

**Stable cell lines**

To generate stable KLHL17^wt^, KLHL17^P234L^, or empty vector expression cell lines, different appropriate vectors were respectively transient transfection into Hela cells by using lip 8000 (Beyotime, Shanghai, China), 48 h later, puromycin (Solarbio, Beijing, China) was added into medium (final concentration: 1 ug/ml), 2-3 weeks later, single clone was picked and cultured.

**Structural analysis of KLHL17^P234L^**

The amino acid sequence of KLHL17^wt^ was obtained from the Universal Protein Resource (Uniprot, <https://www.uniprot.org/uniprotkb/Q6TDP4/entry>), KLHL17’s structure and homology modeling of KLHL17^P234L^ were predicted by using AlphaFold 3 (https://golgi.sandbox.google.com/), and were visualized in 3D using PyMOL software 2.3.0.

**Pathogenicity interpretation of KLHL17^P234L^**

Online REVEL (https://sites.google.com/site/revelgenomics/) and CADD (https://cadd.gs.washington.edu/) tools were used for predicting the pathogenicity of KLHL17^P234L^.

**Reverse transcription reaction and quantitative real-time PCR**

Total RNA was extracted from cultured cells using TRIzol, according to the manufacturer’s instructions (Invitrogen, Cat.#15596018, USA). Reverse transcription reaction and real-time PCR were carried out as we previously described ^2^. The relative expression of *klhl17* was calculated with the 2^-ΔΔCt^ method as we previously described^2^. Primers are listed in Table S1.

**Western blot**

Total protein (30 μg) extracted from samples were separated electrophoretically on 10 % SDS-PAGE gels (Epizyme, PG112, Shanghai, China) and transferred electrophoretically to PVDF membranes (Merck, Cat.#IPVH00010, German). Membranes were blocked using QuickBlock™ Blocking Buffer (Beyotime, Cat.#P0220, Shanghai, China) for 15min at room temperature, followed by incubation with primary antibodies specific for Anti-flag (1:2000, Mouse, Fitgene, Cat.#FI01104s, Guangzhou, China), Anti-KLHL17 (1:2000, Rabbit, Abcam, Cat.#ab122164, England), PI3K-p110 (1:300, Rabbit, Proteintech, Wuhan, China), AKT (1:5000, Mouse, Proteintech, Wuhan, China), p-AKT (Ser473, 1:10000, Rabbit, Proteintech, Wuhan, China), or Anti-β-actin (1:5000, Mouse, Proteintech, Cat.#81115-1-RR, Wuhan, China) overnight at 4 °C; then were incubated with Anti-Mouse IgGκ-HRP (1:10000, Santa Cruz Biotechnology, Cat.#sc-516102, Shanghai, China) or Anti-Rabbit IgG (H+L) HRP (1:10000, Emarbio Science & Technology Co., LTD, Cat.#S0001, Beijing, China) for 2 h at room temperature. Membranes were visualized using enhancedchemiluminescence (Vazyme, Cat.#E411-0405, Nanjing, China), photographed using a ProteinSimple Fluorchem M System (ProteinSimple, USA). β-actin was selected as the internal reference protein.

**Measurement of cell proliferation.**

Cell-counting kit-8 (CCK8, UElandy, Cat.#C6005M, Suzhou, China) was used to detect cell proliferation, according to the manufacturer’s instructions. Briefly, cells (80–90% confluent) were seeded in 96-well plates, transfected with different vectors, then the cell proliferation capacity was detected at an OD of 450 nm using a microplate reader (Varioskan™ LUX, Thermo, USA).

**Effect of inhibitor GDC-0941 on the cell proliferation**

SH-SY5Y cells (80–90% confluent) were seeded in a 96-well plate, transfected with different vectors, 24 h later, cells were treated with DMSO or PI3K inhibitor GDC-0941 (5 μM, MCE, Cat.HY-20180, USA) for 6 h, then the midium was refreshed, CKK8 assay was performed at 72 h post-transfection.

**Cell migration assay**

Wound healing test was used to detect the migration ability of Hela cells. Transfected Hela cells were seeded in 6-well plates (5-7×10^5^ cells per well), 24 h later, scratched with a sterile 10 µl pipette tip, the cells were washed with PBS and added 2 ml fresh medium containing 1% FBS. Images of the wound area were recorded with a fluorescence microimaging system (Olympus IX73, Japan) at 0 h and 48 h after scratching. Images were analyzed using ImageJ (NIH). Cell migration rate (%) was calculated as (the distance of cell migration / the distance of the initial wound) × 100 ^3^.

**Immunofluorescence**

Cells were fixed with 4% paraformaldehyde for 20 min, followed by permeabilization with 0.5% Triton X-100 for 10 min, incubated with YF^®^594-Phalloidin (UElandy, Cat.# YP0052S, Suzhou, China) at a dilution of 1:100 for 20 min at room temperature. Samples were counterstained with DAPI (UElandy, Cat.#A4084, Suzhou, China) and photographed using a fluorescence microimaging system (Olympus IX73, Japan).

**Animal model**

C57BL/6J mice (6-8 weeks) were purchased from the Hunan SJA Laboratory Animal Co., Ltd (Hunan, China) and were maintained in a pathogen-free facility. The protocol was approved by Guilin Medical University Ethics Committee. Newborn mice were obtained by planned crossed adult mice; the pregnant dams were monitored daily to confirm the age of the pups. As shown in Fig. S3, postnatal 0-1day (P0-1) mice (1.2-1.5 g) were randomly divided into three groups (n=9 per group): KLHL17^wt^ vector group, KLHL17^P234L^ vector group, and empty vector group; then were anesthetized on ice for 1-3 min, 2 μl lentivirus (titer: 2×10^5^ TU/ml) was slowly administered to newborn mice via intracerebroventricular (ICV) injection. Notably, in order to simulate the actual situation of KLHL17^P234L^ expression in vivo, we did not adjust the lentivirus injection dose to make equal expression levels of KLHL17 and KLHL17 ^P234L^ in mice. The injection site was identified at 2/5 of the distance from the lambda suture to each eye, the injection depth was 3 mm; slowly removed the needle and waited the first injection site to close before injecting the contralateral ventricle; when the mouse was fully recovered it was placed back into cage along with its mother ^4^. All the newborn mice were examined daily to evaluate their survival statue. 120 days later, mouse behavior tests were performed to evaluate the role of KLHL17 and its variant, KLHL17^P234L^, in brain function.

All experimental mice were habituated in the behavior room for one week prior to undertaking behavior tests.

Elevated plus maze test (EPMT) was performed to assess anxiety-like behavior in mice using a maze consisting two enclosed arms (30 × 5 × 15 cm) and two open arms (30 × 5 cm) that elevated to a height of 40 cm above the floor ^5^. Mice were individually placed in the center area of the maze facing one open arm and allowed to freely explore (Fig. 1J); their movement was recorded for 5 min with videotaping. Time spent in the closed arms and the open arms were quantified with the smart video tracking system (ANY-maze 7.20，USA).

Open field test (OFT) was performed to assess exploratory activity and anxiety-like behavior in mice ^5^. Mice were individually placed in the center of the open box (50 × 50 × 39 cm, SA215, SansBio, China) to freely explore (Fig. 1K). Moving distance and spending time by each mouse were recorded for 5 min with the smart video tracking system (ANY-maze 7.20，USA).

Y-Maze test (YMZT) was performed to assess exploratory behavior, spatial learning and working memory in mice using a 3-armed Y-Maze apparatus (35 ×5 × 25 cm, 120°, SA204, SansBio, China) ^5-7^. Mice were individually placed in the arm designated (A) and allowed to freely explore for 5 min with videotaping (Fig. 1L). Spontaneous alternation behavior (%) was calculated as (number of correct alternations/total number of arm entries minus two) × 100 ^4^.

**Necropsy, sample, histology**

The animals were euthanized with intraperitoneal sodium pentobarbital, and the brains were quickly prepared for quantitative real-time PCR and histological analysis.

Hematoxylin and eosin (H&E)-staining: The brains were fixed, embedded in paraffin, and sectioned into 3-µm thick; sections were dewaxed, hydrated, and stained with H&E solution. Morphological changes in the hippocampal region were observed using SLIDEVIEW VS200 Research Slide Scanner (Olympus, Japan). Nissl staining: The tissue sections were dewaxed, hydrated, and stained with toluidine blue; they were differentiated, dehydrated, transparent, sealed and subsequent Nissl bodies observed. Morphological changes in the hippocampal region were observed using SLIDEVIEW VS200 Research Slide Scanner (Olympus, Japan). and the numbers of Nissl bodies were measured using ImageJ (NIH).

Luxol fast blue (LFB) staining was performed to assess myelination: The paraffin sections were de-waxed, rehydrated and incubated in LFB staining buffer at 60℃ for 1 h, differentiated in Li2CO3 buffer and counterstained with eosin staining solution, then dehydrated in anhydrous ethanol and made transparent with xylene. Morphological changes in the hippocampal region were observed using SLIDEVIEW VS200 Research Slide Scanner (Olympus, Japan).

Immunocytochemistry (IHC): The tissue sections were performed as we previously described^2^. Briefly, mouse brain samples were incubated with antibodies against flag (1:200, Mouse, Fitgene, Cat.#FI01104s, Guangzhou, China) as the primary antibody, and then with biotinylated secondary antibody (1:200, Santa Cruz Biotechnology, Cat.#sc-516102, Shanghai, China). Hematoxylin was used as a tissue counterstain.

**Statistical analysis**

Data are presented as the means ± standard deviation. To compare multiple groups, the data were analyzed by one-way or two-way analysis of variance (ANOVA) followed by Tukey’s post hoc test if data were normally distributed or by the Kruskal–Wallis test followed by Mann-Whitney U test if the data were non-normally distributed. Difference was considered statistically significant at P < 0.05.

**Reference**

1. Kalidasan V, Ng WH, Ishola OA, Ravichantar N, Tan JJ, Das KT. A guide in lentiviral vector production for hard-to-transfect cells, using cardiac-derived c-kit expressing cells as a model system. *Sci Rep*. Sep 28 2021;11(1):19265. doi:10.1038/s41598-021-98657-7

2. Huang B, Feng Z, Zhu L, et al. Silencing of MicroRNA-503 in Rat Mesenchymal Stem Cells Exerts Potent Antitumorigenic Effects in Lung Cancer Cells. *Onco Targets Ther*. 2021;14:67-81. doi:10.2147/ott.S282322

3. Zhu J, Wang Y, Li D, Zhang H, Guo Z, Yang X. Interleukin-35 promotes progression of prostate cancer and inhibits anti-tumour immunity. *Cancer Cell Int*. 2020;20:487. doi:10.1186/s12935-020-01583-3

4. Massaro G, Mattar CNZ, Wong AMS, et al. Fetal gene therapy for neurodegenerative disease of infants. *Nature Medicine*. 2018/09/01 2018;24(9):1317-1323. doi:10.1038/s41591-018-0106-7

5. Yoshizaki K, Asai M, Hara T. High-Fat Diet Enhances Working Memory in the Y-Maze Test in Male C57BL/6J Mice with Less Anxiety in the Elevated Plus Maze Test. *Nutrients*. Jul 9 2020;12(7)doi:10.3390/nu12072036

6. Cleal M, Fontana BD, Ranson DC, et al. The Free-movement pattern Y-maze: A cross-species measure of working memory and executive function. *Behav Res Methods*. Apr 2021;53(2):536-557. doi:10.3758/s13428-020-01452-x

7. Kim J, Kang H, Lee YB, Lee B, Lee D. A quantitative analysis of spontaneous alternation behaviors on a Y-maze reveals adverse effects of acute social isolation on spatial working memory. *Sci Rep*. Sep 7 2023;13(1):14722. doi:10.1038/s41598-023-41996-4

**Figure S1** Computerized analysis of the KLHL17 structure. **(A)** 3D structure of KLHL17 using PyMOL software 2.3.0. **(B)** Schematic structure of KLHL17 and the mutation (red arrow) localizing in the BACK domain, a highly conserved region.

**Figure S2** Levels of mRNA and protein expression of KLHL17 in KLHL17^P234L^ doubled transient transfection. **(A, B)** Quantitative real-time PCR (A) and western blot analysis (B) of KLHL17 expression in cells transfected with different vectors (72 h after transfection). **(C)** CCK8 assay showed the effects of KLHL17 overexpression with different vectors (72 h after transfection) on the proliferation ability of Hela and SH-SY5Y cells. **(D)** The wound healing assay showed the effects of KLHL17 overexpression with different vectors on the migration ability of Hela cells (bar: 200 μm). Flag, 3xflag-tagged empty vector control; WT, 3xflag-tagged KLHL17^wt^ vector; MUT, 3xflag-tagged KLHL17^P234L^ vector.

**Figure S3** CCK8 assay showed the effects of small molecule inhibitor GDC-0941 on SH-SY5Y cell proliferation (72 h after transfection). Two-way ANOVA with Tukey's post hoc test for multiple comparisons was applied. ^*^*P* < 0.05, ^**^*P* < 0.01, ^***^*P* < 0.001, ^****^*P* < 0.0001; ns, no significance.

**Figure S4** The schematic diagram of the animal experiment. Briefly, post-natal 0–1 day (P0–1) mice (1.2–1.5 g) were randomly divided into three groups (*n* = 9 per group): KLHL17^wt^ vector group, KLHL17^P234L^ vector group, and empty vector group; then, the mice were anesthetized on ice for 1–3 min, 2 μL lentivirus (titer: 2 × 10^5^ TU/mL) was slowly administered to newborn mice via intracerebroventricular injection. Notably, to simulate the actual situation of KLHL17^P234L^ expression *in vivo*, we did not adjust the lentivirus injection dose to make equal expression levels of KLHL17 and KLHL17^P234L^ in mice. All the newborn mice were examined daily to evaluate their survival status. 120 days later, exogenous human KLHL17 expression levels in the hippocampus region were detected by quantitative PCR and immunohistochemistry assay; hippocampal neuronal morphology was visualized by Luxol fast blue staining, hematoxylin & eosin staining, and Nissl staining; motor, learning, memory, and cognition were evaluated by a series of behavior assays. Flag, 3xflag-tagged empty vector-administrated mice; WT, 3xflag-tagged KLHL17^wt^ vector-administrated mice; MUT, 3xflag-tagged KLHL17^P234L^ vector-administrated mice. The figure was created with BioRender.com.

**Figure S5** Immunohistochemical staining of Flag proteins in the hippocampal CA1 area of the mice intracerebroventricularly administrated with different lentivirus for 120 days. Normal, age-matched normal mice without any treatment; Flag, 3xflag-tagged empty vector-administrated mice; WT, 3xflag-tagged KLHL17^wt^ vector-administrated mice; MUT, 3xflag-tagged KLHL17^P234L^ vector-administrated mice.

**Figure S6** Quantitative real-time PCR for KLHL17 mRNA expression in the hippocampus of the mice intracerebroventricularly administrated with different lentivirus for 120 days. ^*^*P* < 0.05, ^**^*P* < 0.01; ns, no significance. Flag, 3xflag-tagged empty vector-administrated mice; WT, 3xflag-tagged KLHL17^wt^ vector-administrated mice; MUT, 3xflag-tagged KLHL17^P234L^ vector-administrated mice.
