# Supplementary material for: A novel mutation in the KLHL17 gene is associated with neurodevelopmental disorders
Source: Genes Dis. 2025 Jan 10;12(5):101528. doi: 10.1016/j.gendis.2025.101528 (PMC12164030; doi:10.1016/j.gendis.2025.101528)
Supplement: Multimedia component 2 [file mmc2.docx]

**Supplementary table 1. Primers for vector construction and quantitative real time PCR.**

| **Primer name** | **Sequence** |
| --- | --- |
| Vector construction | |
| KLHL17^wt^- Forward | AAGGATGACGATGACAAGGAATTCATGCAGCCCCGCAGCGAG |
| KLHL17^wt^- Reverse | GATCCTTGCGGCCGCGGATCCTCAGAGGCTGGTGGAGGA |
| KLHL17^P234L^- Forward | GCTGCTGCTCCTGAAACAGGTTCTGGAACT |
| KLHL17^P234L^- Reverse | TGTTTCAGGAGCAGCAGCATAAACTCCTCG |
| Quantitative real time PCR | |
| KLHL17^P234L^- Forward | GCTGCAAGTTTCTACTTGAGTCA |
| KLHL17^P234L^- Reverse | TCAGGCTGTCGCTAGAGACC |
| GAPDH- Forward | GACCACTTTGTCAAGCTCATTTCC |
| GAPDH- Reverse | GTGAGGGTCTCTCTCTTCCTCTTGT |
